# Discovering and Characterizing of Survivin Dominant Negative Mutants With Stronger Pro-apoptotic Activity on Cancer Cells and CSCs

**DOI:** 10.3389/fonc.2021.635233

**Published:** 2021-03-31

**Authors:** Wei Guo, Xingyuan Ma, Yunhui Fu, Chang Liu, Qiuli Liu, Fabiao Hu, Hui Miao, Tong Zhang, Yuping Liu, Myong Hun Han, Fang You, Yi Yang, Wenyun Zheng

**Affiliations:** ^1^ State Key Laboratory of Bioreactor Engineering, East China University of Science and Technology, Shanghai, China; ^2^ Shanghai Key Laboratory of New Drug Design, School of Pharmacy, East China University of Science and Technology, Shanghai, China; ^3^ Department of Chemical and Biomolecular Engineering, National University of Singapore, Singapore, Singapore; ^4^ SinGENE Biotech Pte Ltd, Singapore Science Park, Singapore, Singapore

**Keywords:** survivin mutant (TmSm34/84), A549 cells and CSCs, discovering and characterizing, stronger pro-apoptotic activity, sensitizing chemotherapeutic drugs

## Abstract

Survivin as a member of the inhibitor of apoptosis proteins (IAPs) family is undetectable in normal cells, but highly expressed in cancer cells and cancer stem cells (CSCs) which makes it an attractive target in cancer therapy. Survivin dominant negative mutants have been reported as competitive inhibitors of endogenous survivin protein in cancer cells. However, there is a lack of systematic comparative studies on which mutants have stronger effect on promoting apoptosis in cancer cells, which will hinder the development of novel anti-cancer drugs. Here, based on the previous study of survivin and its analysis of the relationship between structure and function, we designed and constructed a series of different amino acid mutants from survivin (TmSm34, TmSm48, TmSm84, TmSm34/48, TmSm34/84, and TmSm34/48/84) fused cell-permeable peptide TATm at the N-terminus, and a dominant negative mutant TmSm34/84 with stronger pro-apoptotic activity was selected and evaluated systematically *in vitro*. The double-site mutant of survivin (TmSm34/84) showed more robust pro-apoptotic activity against A549 cells than others, and could reverse the resistance of A549 CSCs to adriamycin (ADM) (reversal index up to 7.01) by decreasing the expression levels of survivin, P-gp, and Bcl-2 while increasing cleaved caspase-3 in CSCs. This study indicated the selected survivin dominant negative mutant TmSm34/84 is promising to be an excellent candidate for recombinant anti-cancer protein by promoting apoptosis of cancer cells and their stem cells and sensitizing chemotherapeutic drugs.

## Introduction

Cancer is the second leading cause of death globally, killing 9.6 million people worldwide in 2018 alone (1, 2). The conventional cancer therapy is surgery followed by radiotherapy and chemotherapy. However, radiotherapy and chemotherapy will also damage normal tissue cells when they do harm to tumor cells (3). So molecular targeted therapies emerged as the times require, which selectively kill tumor cells through tumor specific markers while show limited or non-existent side effects on body normal cells (4). Survivin, a member of inhibitor of apoptosis proteins (IAPs) family, plays an important role both in inhibiting apoptosis and regulating mitosis of cells (5, 6). Its overexpression in cancer cells and cancer stem cells (CSCs) is associated with tumor development, metastasis, and even drug resistance, so survivin is an attractive molecular target for cancer therapy (7, 8).

Many strategies have been developed to damage the function of survivin, including small molecule inhibitors, antisense oligonucleotides (ASO), small interfering RNA (siRNA) (9). Despite some achievements in suppressing the activity of cancer cells, they may not be suitable for better targeting survivin because they work only by transcriptional inhibition and cannot abolish the function of survivin protein has been existed (10, 11). While another strategy named dominant negative mutants can compete with wild-type survivin protein, thus blocking the biological function of survivin at the terminal of protein expression (12). A recombinant dominant negative protein TATm-survivin (T34A) (TmSm34) was previously obtained by our laboratory, which consisted of a cell-permeable TATm peptide (YARKARRQARR) and a survivin mutant (the 34^th^ Thr was replaced by Ala) (13). TmSm34 could induce apoptosis of most cancer cell lines and enhance the sensitivity of breast cancer cell lines, such as T47-D, MCF-7, and Bcap-37 to adriamycin (ADM) (13, 14). Moreover, TmSm34 was proved to inhibit the growth and induce apoptosis of breast CSCs (15). However, TmSm34 may not show anti-cancer activity ideally. A TmSm protein with better anti-cancer activity needs to be found to achieve the goal of inhibiting survivin’s activity more efficiently.

**Figure 1:**
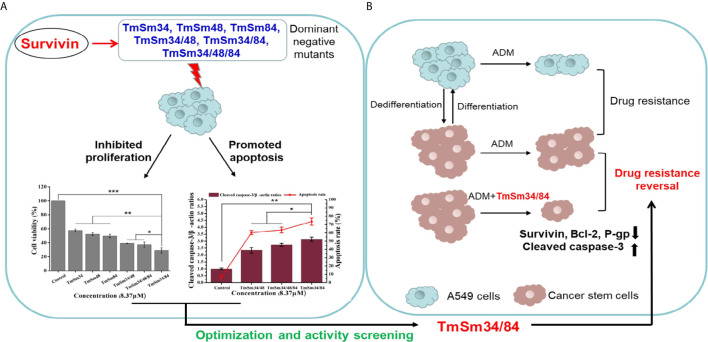
Systematical diagram of design idea and researching flow of discovering a dominant negative mutant derived from survivin with stronger pro-apoptotic activity to cancer. **(A)** Compared with TmSm34, TmSm48, TmSm84, TmSm34/48, and TmSm34/48/84, TmSm34/84 had the strongest capacity in inhibiting proliferation and promoting apoptosis of A549 cells. **(B)** TmSm34/84 could reverse the drug resistance of A549 CSCs to Adriamycin (ADM) by down-regulating survivin, Bcl-2, and P-gp expression, while up-regulating cleaved caspase-3 expression.

Survivin dominant negative protein C84A which fused with a cell-permeable poly-arginine (R9) peptide was also shown to be capable of inducing apoptosis of cancer cells (16). Besides, HeLa cells stably overexpressing survivin mutant T48A showed elevated caspase-3 activity compared with cells expressing survivin-GFP, indicating that T48A could induce apoptosis of HeLa cells (17). Interestingly, Zhang et al. constructed a double-site survivin mutant TC34/84AA which expressed by adenoviruses showed more potent capacity in inhibiting the growth of hepatocellular cancer cells compared with single-site mutant T34A or C84A alone (10, 18). The results from zhang gave us a hint that we could mutate some other sites based on TmSm34 to obtain a TmSm protein with stronger capacity in inducing apoptosis on cancer cells. So, in this study, we designed and constructed a series of different amino acid mutants from survivin (TmSm34, TmSm48, TmSm84, TmSm34/48, TmSm34/84, and TmSm34/48/84) fused cell-permeable peptide TATm at the N-terminus, and systematically analyzed the anti-cancer viability of various TmSm proteins *in vitro* for discovering a dominant negative mutant with stronger pro-apoptotic activity to cancer cells. The overall diagram of design idea and researching flow was shown in **Figure 1**. By this study, the selected survivin dominant negative mutant is promising to be an excellent candidate for recombinant anti-cancer protein by promoting apoptosis of cancer cells and their stem cells and sensitizing chemotherapeutic drugs.

## Materials and Methods

### Materials

*E. coli* BL21 (DE3), *E. coli* DH5α, pET-24a (+) were provided by Invitrogen (CA, USA). pET-24a (+)-TmSm34, pET-24a(+)-TmSm48, and pET-24a(+)-TmSm84 were previously constructed by our laboratory. MTT were supplied by Solarbio (Beijing, China). Hoechst 33342 were purchased from Yeasen (Shanghai, China). All other reagents were of analytical grade and acquired from Sigma-Aldrich (Shanghai, China). Human lung cancer cell A549 and normal liver cell L-02 was obtained from the Type Culture Collection Committee of Chinese Academy of Science (Shanghai, China). Fetal bovine serum (FBS), penicillin/streptomycin (10,000 U/ml penicillin and 10 mg/ml streptomycin), Roswell Park Memorial Institute 1640 medium (RPMI1640), Dulbecco’s Modified Eagle medium (DMEM), DMEM/Nutrient Mixture F-12 medium (DMEM/F12), and B27 were purchased from Gibco (Waltham, USA). Basic fibroblast growth factor (bFGF) and epidermal growth factor (EGF) were acquired from Pepcech (Cathy, USA).

### Plasmid Construction and Protein Expression

Based on pET-24a(+)-TmSm34, the codon ACT corresponding to T48 and the codon TGC corresponding to C84 were mutated to the codon GCC corresponding to Ala by overlapping extension PCR technology, and obtained TmSm34/48 and TmSm34/84 genes, respectively. Then, pET-24a (+)-TmSm34/48 was used to amplify TmSm34/48/84 gene. **Table S1** shows the primer sequences. The PCR amplification fragments were purified, cleaved by *Nde* I and *Xho* I, and ligated into pET-24a (+) plasmid. Finally, the expression vectors were transferred into *E. coli* BL21 (DE3) and induced with 0.75 mM IPTG for expression. Due to have His tag, TmSm proteins were purified by nickel column. The eluted solution was renatured in dialysate with a gradient of Urea (4, 2, 1, and 0 M) in buffer A (20 mM PB, 500 mM NaCl, 5% Glycerol, pH 7.0) at 4°C. Finally, the refolded protein was concentrated by 3 kDa ultrafilter tubes. Protein expression and concentration were identified by SDS-PAGE and Bradford kit respectively.

### Cell Culture

A549 and L-02 cells were respectively cultured in RPMI 1640 and DMEM, supplemented with 10% (v/v) FBS and 1% streptomycin-penicillin, and then incubated in an atmosphere with 5% CO_2_ at 37°C. To isolate CSCs from A549 cells, A549 cells (2 × 10^4^ cells/well) were plated in six-well ultra-low attachment plates (Corning, USA) in serum-free DMEM/F12, containing 10 ng/ml bFGF, 20 ng/ml EGF, 2% B27, and 1% streptomycin-penicillin. Once the spheres formed, replaced the medium every 3 days by letting them settle to the bottom by gravity and passage the cells every 7 days until the third-generation spheres were obtained.

### Cytotoxicity Studies

The viability of cells and IC_50_ of drugs were detected by MTT assay. A549 cells, L-02 cells, and CSCs (1 × 10^4^ cells/well) were seeded onto 96-well plates and incubated with TmSm proteins, ADM, or their combination. After culturing for 24 h, MTT solution (5 mg/ml) was added into each well and incubated at 37°C for 4 h. Subsequently, the supernatant in the well was carefully removed and 150 μl dimethyl sulfoxide (DMSO) was added to dissolve the produced formazan. The plate was then read by a microplate reader (Biotek, USA) at 490 nm. IC_50_ was calculated by SPSS 22.0. Resistance index (RI) was the ratio of IC_50_ of CSCs and IC_50_ of A549 parental cells.

### Apoptosis Assay

A549 cells, L-02 cells, and CSCs were resuspended into single cell suspension and plated at a density of 4 × 10^5^ cells/well into six-well plates. After culturing for 24 h, A549 and L-02 cells were treated with TmSm proteins, while CSCs were incubated with TmSm proteins, ADM, and their combination, respectively. Following incubation with drugs for 24 h, cells were harvested by centrifugation at 2,000 rpm for 5 min and washed twice with PBS. Further, cells were resuspended in 100 µl binding buffer. Subsequently, 5 μl Annexin V-FITC and 5 μl propidium iodide (PI) (Elabscience, China) were added to each tube for 30 min incubation in the dark at 25°C to detect the effect of TmSm protein drugs on cell apoptosis. Meanwhile, 5 μl Annexin V-FITC and 5 μl 7-AAD (Elabscience, China) were used to detect the effect of ADM on cell apoptosis. The resultant samples were immediately analyzed by flow cytometry using FITC channel (excitation: 488 nm and emission: 525nm), PI channel (excitation: 535 nm and emission: 615 nm), and 7-AAD channel (excitation: 488 nm and emission: 670 nm).

### Immunofluorescence Assay

A549 cells (4 × 10^5^ cells/well) and CSCs suspension were plated onto confocal petri dish (NEST, China) and cultured for 6 h, respectively. Then, cells were washed with PBS, fixed with 4% paraformaldehyde, and blocked in Tris-buffered saline containing Tween-20 (TBST) with 3% BSA (Solarbio, China). Further, cells were incubated with anti-CD133 (1:250) and anti-CD44 (1:250) primary antibodies (Proteintech, USA) overnight at 4°C. For visualization, cells were then stained with PE-conjugated (1:100) and FITC-conjugated (1:100) secondary antibodies (Proteintech, USA) in the dark at 25°C for 1 h, respectively. Finally, the nuclei were labeled with Hoechst 33342 (Aladdin, China). Images were captured under a fluorescent microscope (Nikon, Japan).

### Fluorescence Intensity Assay

A549 cells and CSCs were digested with 0.25% trypsin, dispersed into suspension by pipetting, and centrifuged at 1,000 rpm for 5 min to collect the cells. Then the cells were washed twice with PBS, and resuspended in 100 μl PBS (1 × 10^7^ cells/ml). Subsequently, 5 μl PE anti-human CD133 or FITC anti-human CD44 (Biolegend, China) was added to each tube. After 30 min incubation in the dark at 4°C, cells were washed with PBS and resuspended in 200 μl PBS. Finally, cellular fluorescence intensity was analyzed by flow cytometry using FITC channel (excitation: 488 nm and emission: 525nm), PI channel (excitation: 535 nm and emission: 615 nm). To set the background fluorescence levels, PE rat IgG1 κ isotype control and FITC rat IgG1 κ isotype control (Biolegend, China) were used as the negative control.

### Cell Growth Curve and Colony Formation Assay

A549 cells and CSCs (2 × 10^3^ cells/well) were plated onto 96-well plates and cultured in RPMI 1640 containing 10% FBS and 1% streptomycin-penicillin. Culturing medium was replaced every 2 days. Cell proliferation viability was measured at specified times (0, 1, 3, 5, and 7 days) by MTT assay. Moreover, A549 cells and CSCs (5 × 10^2^ cells/well) were plated onto six-well plates to detect the cloning capability. Fourteen days later, cells were washed with PBS and fixed with 4% paraformaldehyde. Subsequently, cells were incubated with a Gimas solution (Solarbio, China). Finally, the numbers of colonies (diameter >0.5 mm) were counted.

### Western Blot

Cells were washed twice in PBS and lysed in RIPA buffer (Solarbio, China) with 1 mM phenylmethanesulfonyl fluoride (PMSF). A BCA protein kit was used to quantify protein concentrations (Beyotime, China). Total proteins (30 μg) were separated on 15% SDS-PAGE gels and transferred to polyvinylidene difluoride (PVDF) membranes (Beyotime, China). The membranes were then blocked in TBST with 3% non-fat milk (Sangon Biotech, China) at 25°C for 2 h and incubated with anti-survivin (1:1,000), anti-caspase-3 (1:1,000), anti-Bcl-2 (1:3,000), anti-P-gp (1:3,000), and anti-β-actin (1:5,000) primary antibodies (Proteintech, USA) overnight at 4°C. Next, the membranes were incubated with the appropriate HRP-conjugated secondary antibodies (1:10,000) (Proteintech, USA) at 25°C for 1 h. Protein bands were visualized by ECL chemiluminescence kit (Sangon Biotech, China), and the gray values of the protein bands were analyzed by Image J.

### Docking Simulation of TmSm Proteins With Caspase-9

The crystal structure of caspase-9 (PDB ID: 2AR9) was downloaded from Protein Data Bank (https://www.rcsb.org/). The three-dimensional (3D) structures of various TmSm proteins were obtained by https://zhanglab.ccmb.med.umich.edu/I-TASSER/. Subsequently, the docking simulation of TmSm proteins with caspase-9 were conducted by ZDOCK. Finally, the docking results of TmSm proteins with caspase-9 were analyzed by PDBePISA and visualized by Pymol.

### Statistical Analysis

The data were obtained from at least three independent experiments and expressed as the mean ± SD. All data were analyzed by SPSS 22.0. *P* < 0.05 was statistically significant.

## Results

### Preparation of TmSm Proteins

Recombinant plasmids were successfully constructed as confirmed by liquid colony PCR and DNA sequencing (**Figure S1**). Then, plasmids were transformed into *E. coli* BL21 (DE3). After IPTG induction, six recombinant proteins (TmSm34, TmSm48, TmSm84, TmSm34/48, TmSm34/84, and TmSm34/48/84) were all expressed in the form of inclusion bodies. Subsequently, inclusion bodies were washed twice with wash buffer and obtained purity of more than 74% (**Table S2**). After purified by nickel column (**Figure S2A**), inclusion bodies achieved a purity of more than 95% (**Figure S2B**). Finally, six active TmSm proteins were acquired by renaturing *via* a refolding buffer.

### Screening and Identification of TmSm Proteins

**Figure 2:**
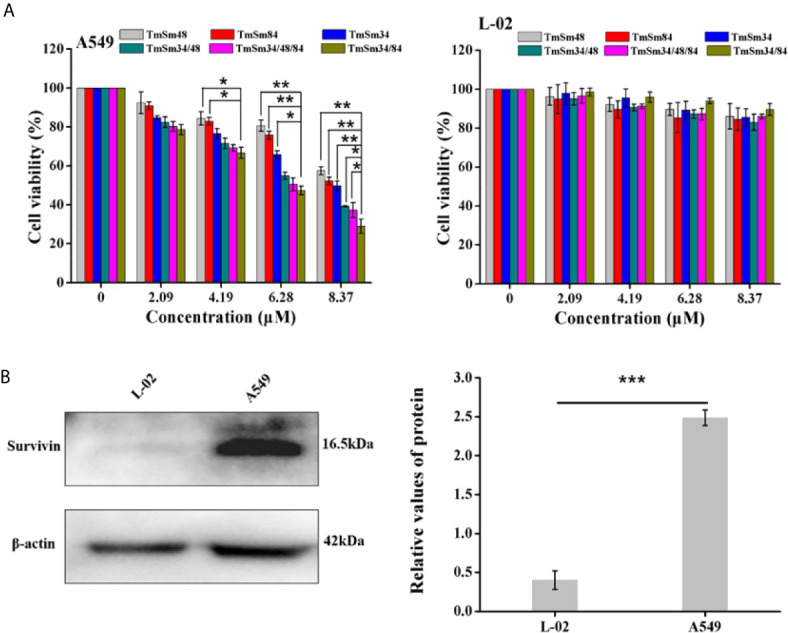
Cytotoxicity assay of TmSm proteins against A549 and L-02 cells. **(A)** The viabilities of A549 and L-02 cells were measured after incubating with different TmSm proteins of the same concentrations gradient (0, 2.09, 4.19, 6.28, and 8.37 μM) for 24 h. **(B)** Survivin expression was determined by Western blot in A549 and L-02 cells. Data were expressed as mean ± SD (*n* = 3). *P < 0.05, **P < 0.01, and ***P < 0.001.

In order to obtain a more efficient TmSm protein, we used MTT, flow cytometry, and Western blot to compare the anti-cancer activity of six TmSm proteins. As revealed in **Figure 2A**, the cell viability of various TmSm proteins against A549 cells all decreased in a concentration-dependent manner. Compared with single-site mutants, multisite mutants had a stronger capacity in inhibiting proliferation of A549 cells. The IC_50_ values of TmSm34, TmSm48, TmSm84, TmSm34/48, TmSm34/84, and TmSm34/48/84 against A549 cells were 9.44, 11.83, 10.20, 6.86, 5.51, and 6.32 μM, respectively (**Table S3**), and TmSm34/84 had the lowest IC_50_ value. Interestingly, the cell viability of TmSm proteins against L-02 cells was above 80%, suggesting that various TmSm proteins did not affect the viability of L-02 cells. The difference of the viability in A549 and L-02 cells after incubating with TmSm proteins might due to the different expression of survivin in A549 cells (2.5-fold) and L-02 cells (0.4-fold) (**Figure 2B**).

**Figure 3:**
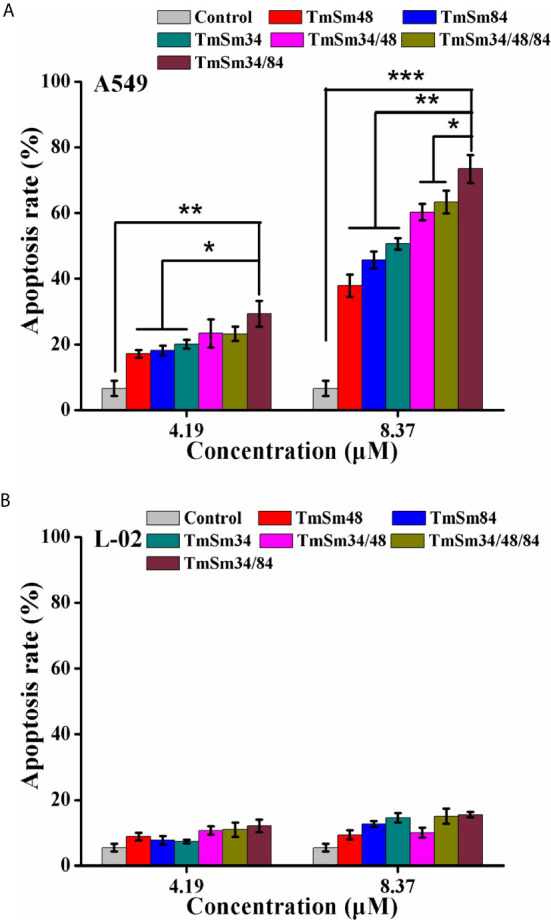
Apoptosis of A549 and L-02 cells were analyzed by flow cytometry. **(A)** A549 cells were incubated with different TmSm proteins of the same concentrations gradient (0, 4.19, and 8.37 μM) for 24 h. Bar diagram depicting the total percentage of apoptotic cells. **(B)** L-02 cells were incubated with different TmSm proteins of the same concentrations gradient (0, 4.19, and 8.37 μM) for 24 h. Bar diagram depicting the total percentage of apoptotic cells. Data were expressed as mean ± SD (*n* = 3). *P < 0.05, **P < 0.01, and ***P < 0.001.

We next investigated the effect of TmSm proteins on the apoptosis of A549 and L-02 cells. As shown in **Figures 3A**, **S3A**, apoptosis was increased in A549 cells treated with TmSm proteins. It was worthy of being noticed that TmSm34/84 with a concentration of 8.37 μM had the strongest ability to induce A549 cells apoptosis than that of other five TmSm proteins (1.45-fold for TmSm34, 1.94-fold for TmSm48, 1.60-fold for TmSm84, 1.22-fold for TmSm34/48, and 1.16-fold for TmSm34/48/84, respectively). However, TmSm proteins showed no significant effect on the apoptosis of L-02 cells (**Figures 3B**, **S3B**), suggesting that TmSm proteins could not induce apoptosis of normal cells with low expression of survivin. Compared with single-site mutants, multisite mutants had a lower IC_50_ value on A549 cells, as well as a more robust capacity in inducing apoptosis of A549 cells, so multisite mutants were chosen for further analysis.

**Figure 4:**
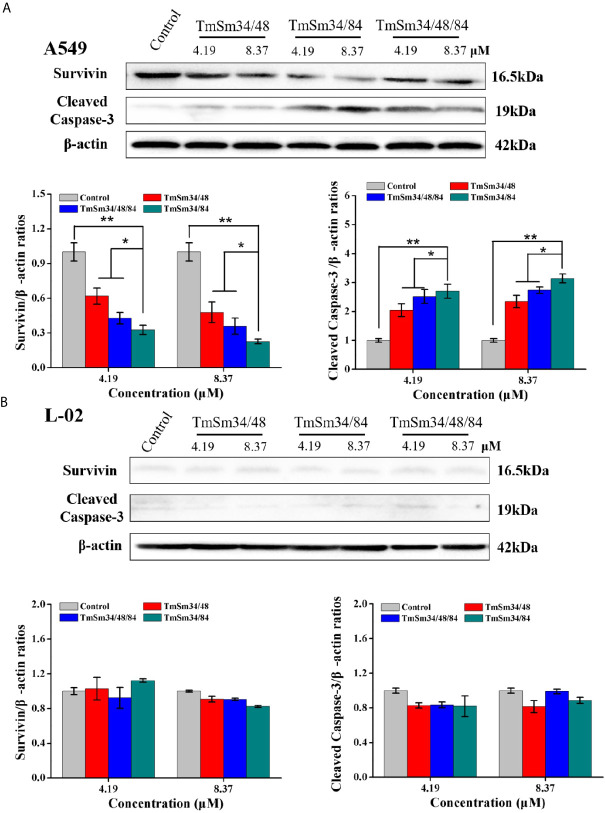
Expression levels of survivin and cleaved caspase-3 in A549 and L-02 cells were determined by Western blot. **(A)** A549 cells were incubated with different TmSm proteins of the same concentrations gradient (0, 4.19, and 8.37 μM) for 24 h, the expression level of survivin and cleaved caspase-3 was detected by Western blot. **(B)** L-02 cells were incubated with different TmSm proteins of the same concentrations gradient (0, 4.19, and 8.37 μM) for 24 h, the expression level of survivin and cleaved caspase-3 was detected by Western blot. Data were expressed as mean ± SD (*n* = 3). *P < 0.05 and **P < 0.01.

Activation of caspase is an essential condition for apoptosis (19). Among them, caspase-3 is in the downstream of the apoptotic response which can degrade a variety of proteins, thus leading to cell apoptosis, and its activation is a sign suggests that apoptosis enters an irreversible stage (18, 20). To further determine whether TmSm34/84 had the strongest ability to promote apoptosis of A549 cells among the three survivin multisite mutants, the expression levels of survivin and cleaved caspase-3 after treating with TmSm proteins were detected by Western blot. Results indicated that TmSm proteins could decrease survivin expression and increase cleaved caspase-3 expression of A549 cells in a dose-dependent manner (**Figure 4A**). Notably, compared with TmSm34/48 and TmSm34/48/84, TmSm34/84 had the strongest ability to down-regulate survivin expression and up-regulate cleaved caspase-3 expression of A549 cells both at concentrations of 4.19 and 8.37 μM (P < 0.05). However, the expression levels of survivin and cleaved caspase-3 in L-02 cells did not occur significantly change (**Figure 4B**). The above results indicated that TmSm34/84 protein was a highly effective anti-cancer candidate drug which had no effect on the viability of normal cells, but had a significant impact on the viability and apoptosis of A549 cells. Unexpectedly, the ability of TmSm34/48/84 to inhibit proliferation and promote apoptosis of A549 cells was weaker than that of TmSm34/84.

### Docking Results for TmSm Proteins With Caspase-9

**Figure 5:**
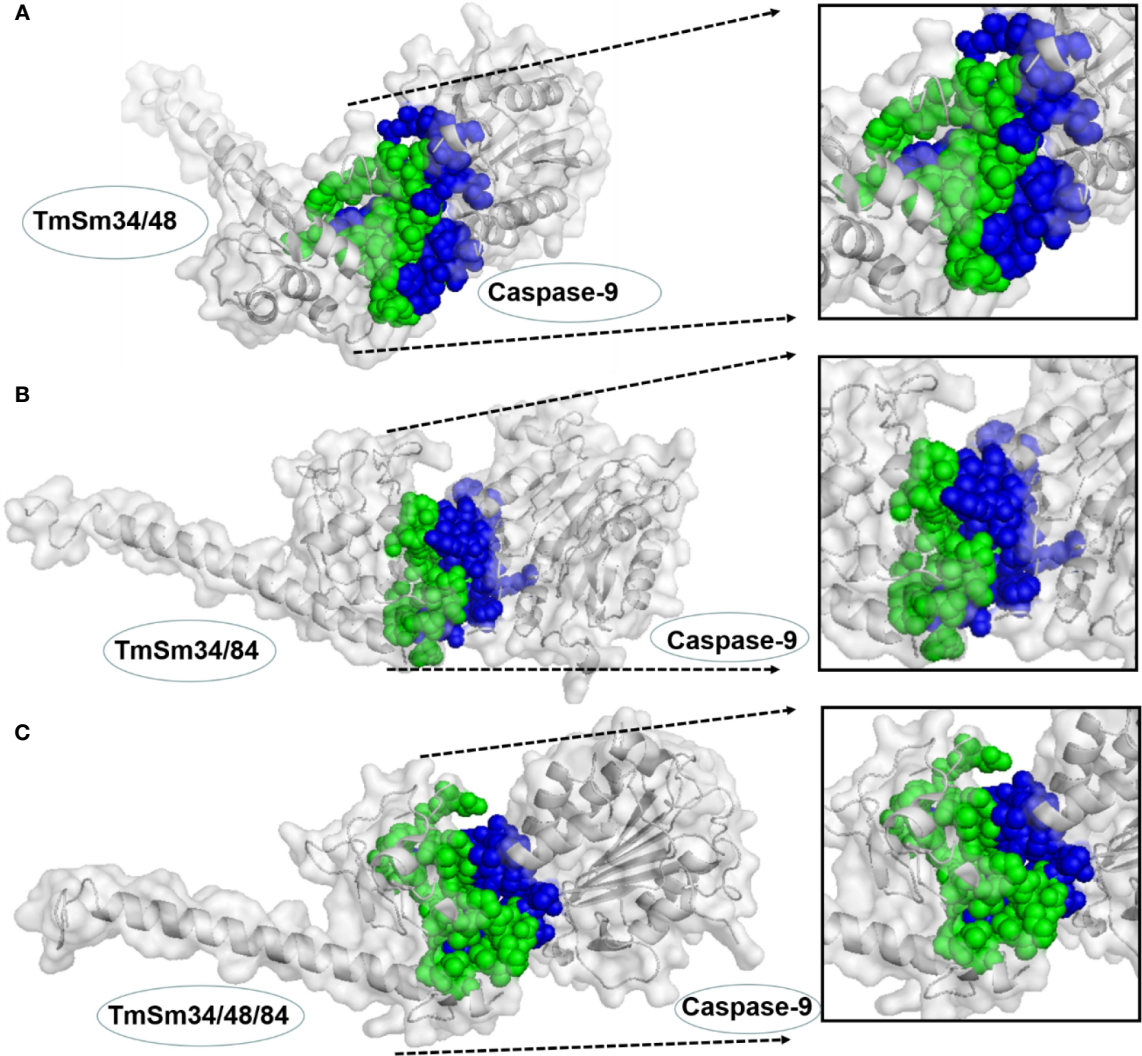
Docking results for different TmSm proteins with caspase-9 were visualized by Pymol. **(A)** Docking results for TmSm34/48 with caspase-9. **(B)** Docking results for TmSm34/84 with caspase-9. **(C)** Docking results for TmSm34/48/84 with caspase-9. The interface areas were highlighted in green and blue.

Survivin functions to inhibit cell apoptosis through suppressing the activity of the apoptotic initiator caspase-9, followed by preventing the activation of the apoptotic effector caspase-3 (21). The TmSm proteins could release the activity of caspase-9 and finally achieve the purpose of promoting cell apoptosis (22, 23). To gain an understanding of the reason that the anti-cancer capacity of TmSm34/48/84 was weaker than that of TmSm34/84, we used ZDOCK to simulate the docking for three multisite TmSm proteins with caspase-9 (**Figure 5**). Subsequently, the interface area and binding free energy of the complex formed by docking were analyzed by PDBePISA. Theoretically, the lower the interface area and the higher the binding free energy are, the less likely the complex will form and the more unstable the structure will be. It could be seen from **Table 1** that the complex interface area formed by the docking of TmSm34/48, TmSm34/84, TmSm34/48/84 with caspase-9 were 1,159.4, 820.5, and 861.4 Å^2^, respectively, and the binding free energy were −16.0, −3.0, and −4.6 kcal/mol, respectively. It is worth noting that, compared with TmSm34/48 and TmSm34/48/84, the docking of TmSm34/84 with caspase-9 had the lowest value for Interface area (820.5 Å^2^) and the highest value for binding free energy (−3.0 kcal/mol), indicating that TmSm34/84 was more difficult to form a complex with caspase-9, thus acquiring a stronger ability to release caspase-9, and eventually showed the highest anti-cancer activity.

**Table 1:** Interface area and binding free energy of TmSm proteins with caspase-9 were analyzed by PDBePISA.

Mutants	Interface area, Å^2^	Δ^i^G, kcal/mol
TmSm34/48	1159.4	−16.0
TmSm34/84	820.5	−3.0
TmSm34/48/84	861.4	−4.6

### Enrichment and Identification of A549 CSCs

**Figure 6:**
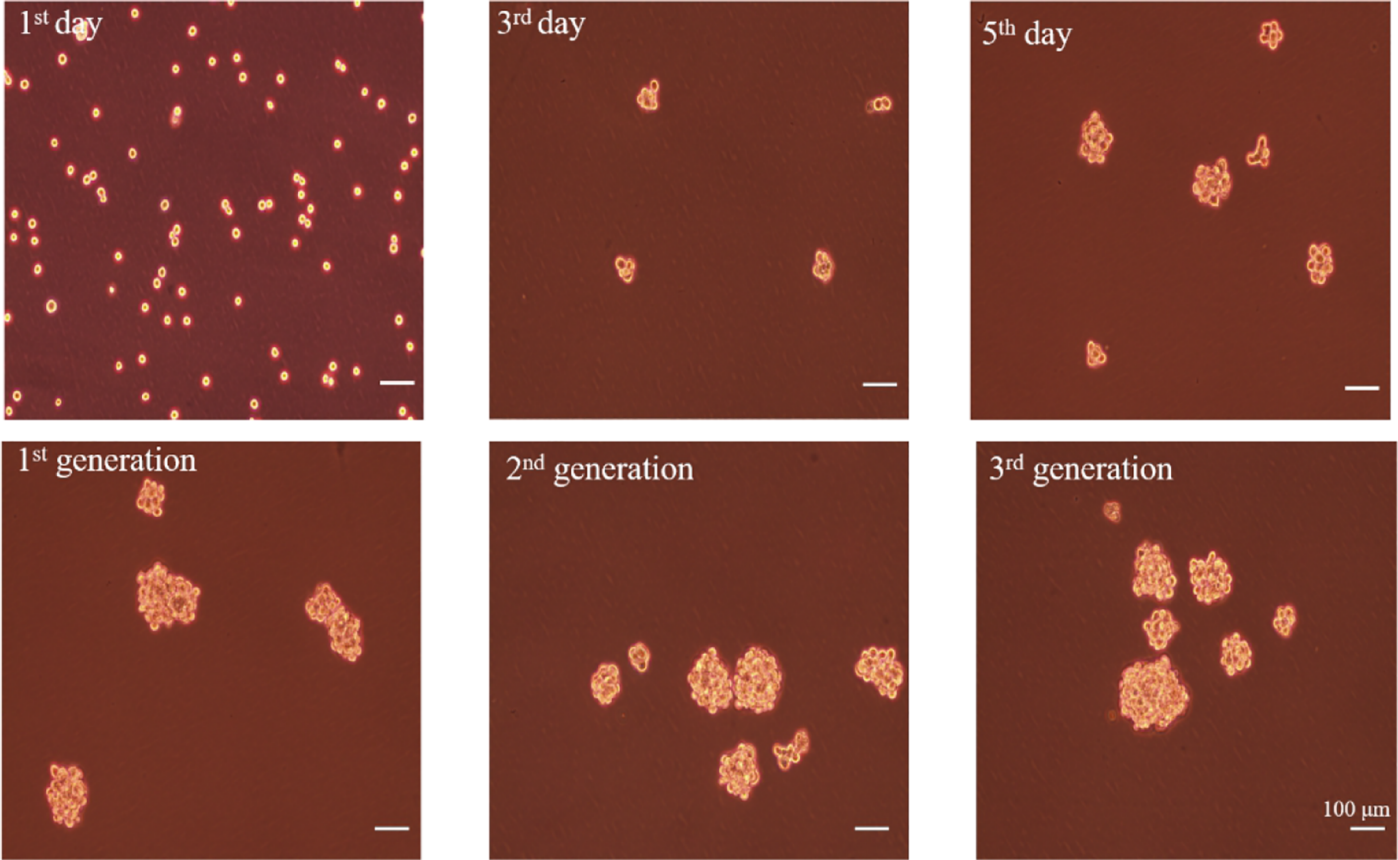
The formation of A549 CSCs induced by serum-free suspension culture. Top, 1-day, 3-day, and 5-day phases of spheres in SFM. Bottom, one-generation, two-generation, and three-generation phases of spheres in SFM. The bar represents a distance of 100 μm.

To enrich A549 CSCs, A549 parental cells were cultured in a defined serum-free medium (SFM) under nonadherent conditions. As shown in **Figure 6**, spheres composed of several single cells were observed after 3 days. About 7 days later, the diameter of the primary spheres reached 150–200 μm. In order to prevent the cell clusters from growing too large, which can lead to necrosis as a result of a lack of oxygen and nutrient exchange at the center of the spheres, passage the cells when primary spheres reach 150 to 200 μm in size. After three passages, spheres became more regular.

**Figure 7:**
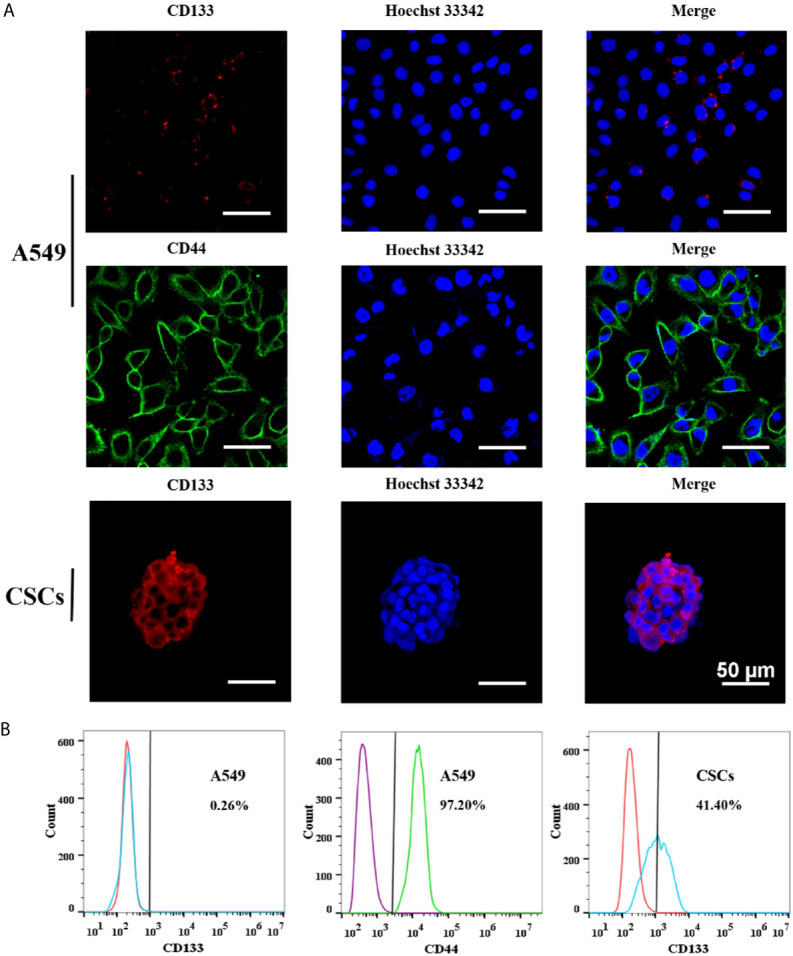
The expressions of stem cell surface markers in A549 CSCs. **(A)** Immunofluorescence staining of A549 cells and CSCs. A549 cells were stained for CD44 and CD133. CSCs were stained for CD133. Pictures were taken using a Nikon microscope. The bar represents a distance of 50 μm. **(B)** Flow cytometry analysis of CD44 expression in A549 cells and CD133 in A549 cells and CSCs. Red histograms presented cells stained with PE Rat IgG1, κ Isotype Control. Blue histograms presented cells stained with PE Anti-Human CD133. Purple histograms presented cells stained with FITC Rat IgG1, κ Isotype Control. Green histograms presented cells stained with FITC Anti-Human CD44.

**Figure 8:**
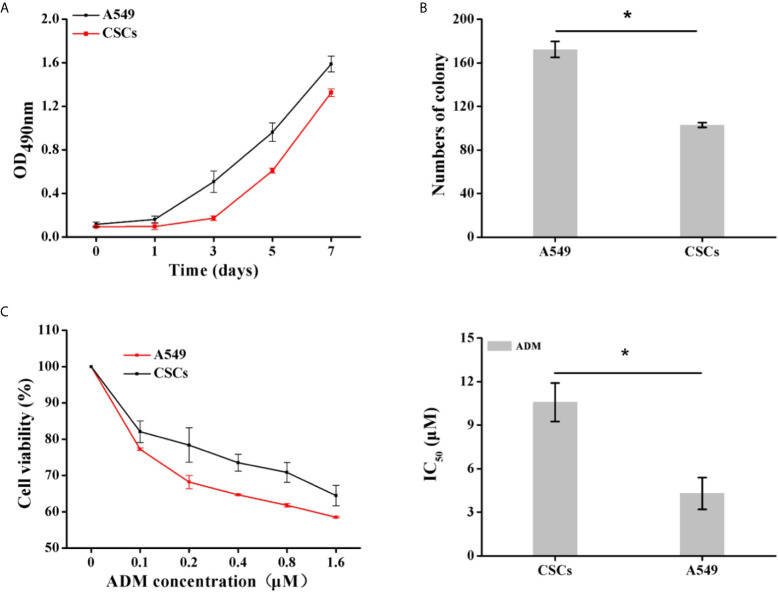
Identification of proliferation capacity and drug resistance of CSCs. **(A)** The growth curve of A549 CSCs based on OD_490nm_ value on days 1, 3, 5, and 7. **(B)** The comparison of colony formation ability between A549 and CSCs for 14 days. **(C)** Viabilities of A549 cells and CSCs after treating with ADM (0, 0.1, 0.2, 0.4, 0.8, and 1.6 μM) for 24 h were determined by MTT assay. Data were expressed as mean ± SD (*n* = 3). *P < 0.05.

To determine whether the spheres cultured in SFM were A549 CSCs, we evaluated the expression of CD133 and CD44 in the cell membrane by immunofluorescence and flow cytometry. CD44 and CD133 were widely accepted as a characteristic of lung CSCs before (24). Immunofluorescence results showed that spheres exhibited higher red fluorescence than A549 parental cells, indicating that spheres had a high CD133 expression (**Figure 7A**). Flow cytometry results further indicated that the proportion of CD133-positive cells in spheres was 41.4%, which was significantly higher than that in A549 parental cells (0.26%) (**Figure 7B**). Interestingly, we found that CD44 was highly expressed in A549 parental cells with a proportion of 97.2%. Therefore, CD44 was not appropriate for identifying A549 CSCs which was in accordance with a previous study (25). Staying at the quiescent stage is also a characteristic of CSCs (26). Thus, the *in vitro* proliferation capacity of spheres was measured. Compared with parental A549 cells, the growth speed of spheres was significantly slow (**Figure 8A**), and the number of colonies formed by spheres was 1.67-fold lower than that of A549 parental cells (**Figure 8B**). Strong drug resistance possession is also an important feature of CSCs, which may be related to the multi-drug resistance of tumor cells (27). ADM is commonly used as a chemotherapeutic drug for lung cancer cure (28). Therefore, the drug resistance of CSCs to ADM was measured by MTT assay. Relative viabilities of A549 parental cells and CSCs after treating with ADM were presented in **Figure 8C**. Compared with A549 parental cells, CSCs demonstrated lower susceptibility to ADM with an IC_50_ value of 10.58 μM, but 4.30 μM for A549 parental cells, suggesting that the RI of CSCs to ADM was 2.46. The above results indicated that spheres formed in SFM were A549 CSCs.

### Increased Expression of Survivin, Bcl-2, and P-gp Is Involved in ADM Resistance of A549 CSCs

**Figure 9:**
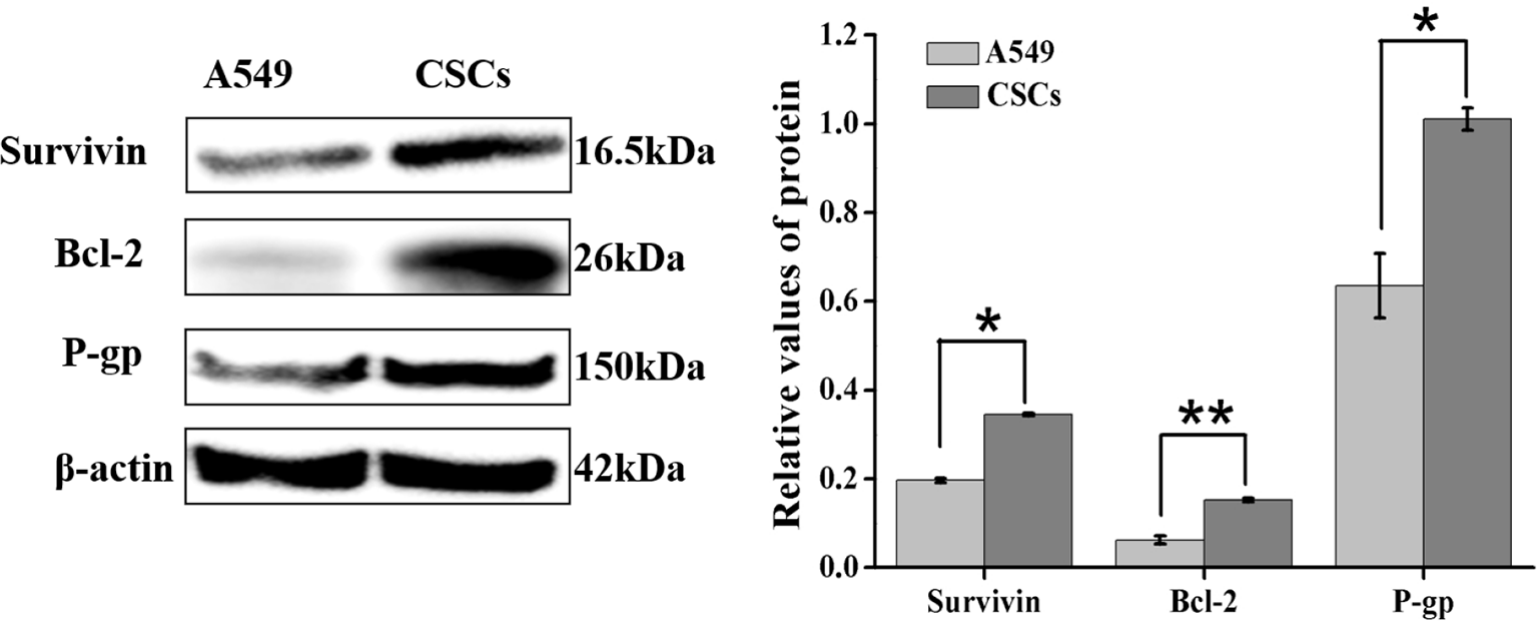
Differential expression of survivin, Bcl-2, and P-gp in A549 parental cells and CSCs were detected by Western blot. Data were expressed as mean ± SD (*n* = 3). *P < 0.05 and **P < 0.01.

To investigate the mechanism for drug resistance of A549 CSCs to ADM, the expression of apoptosis-related proteins (survivin and Bcl-2) and drug resistance-related protein P-glycoprotein (P-gp) in CSCs were analyzed by Western blot. As illustrated in **Figure 9**, the expression levels of survivin, Bcl-2, and P-gp in CSCs were substantially elevated (1.79-fold, 2.43-fold, and 1.58-fold for A549 parental cells, respectively), suggesting that the overexpression of survivin, Bcl-2, and P-gp played an essential role in acquiring drug resistance of CSCs.

### TmSm34/84 Improves the Sensitivity of CSCs to ADM

**Figure 10:**
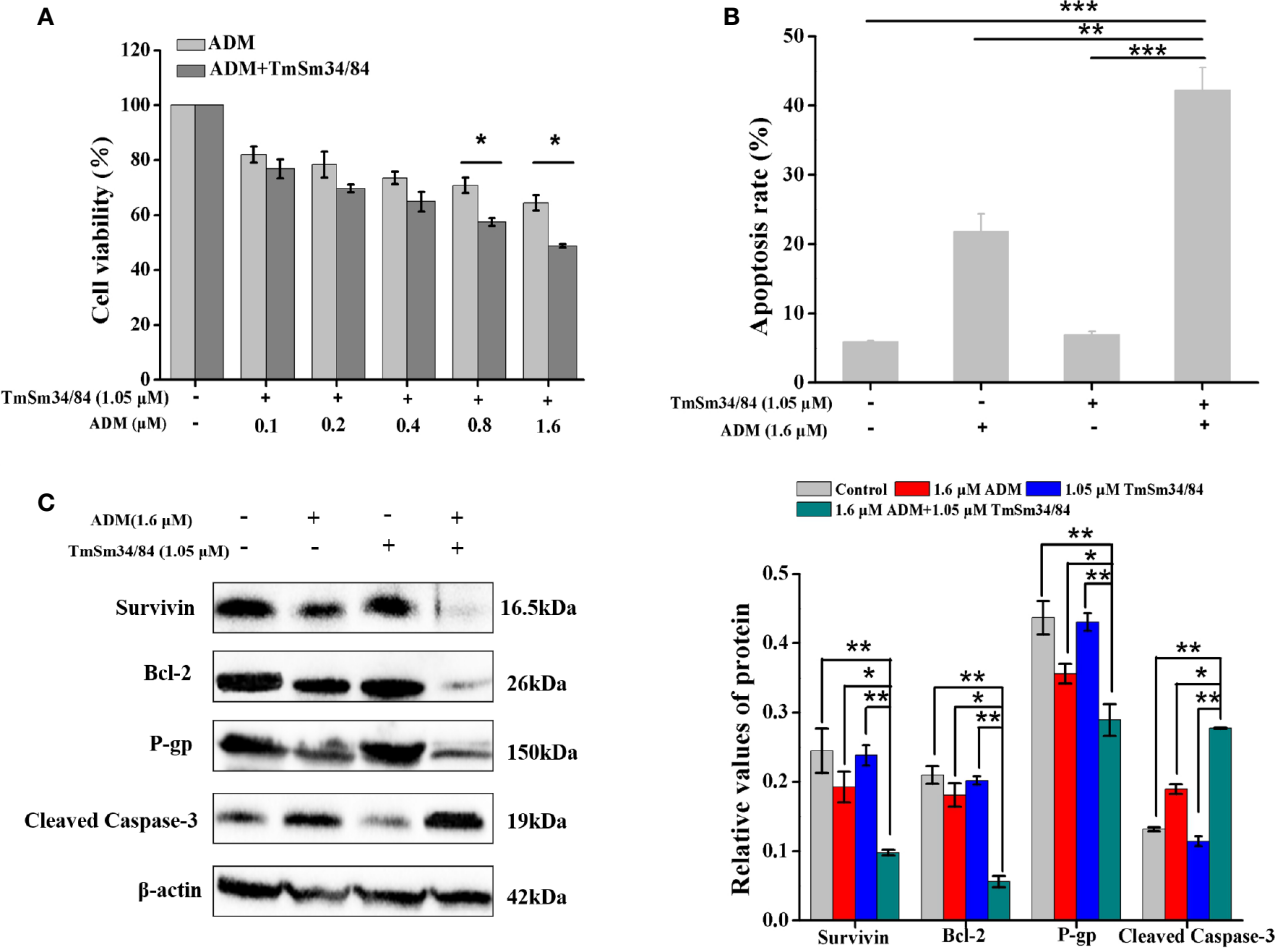
The effect of TmSm34/84 on the drug resistance against CSCs. **(A)** Viability of CSCs was measured by incubating with TmSm34/84 (1.05 μM) plus ADM of different concentrations (0, 0.1, 0.2, 0.4, 0.8, and 1.6 μM) for 24 h, respectively. **(B)** The apoptosis-inducing effect of ADM (1.6 μM), TmSm34/84 (1.05 μM), and their combination on CSCs as tested with flow cytometry, respectively. Bar diagram depicting the total percentage of apoptotic cells. **(C)** The effects of ADM, TmSm34/84, and their combination on the protein expression in CSCs were detected by Western blot, respectively. Data were expressed as mean ± SD (*n* = 3). *P < 0.05, **P < 0.01, and ***P < 0.001.

To further verify the cancer suppressive activity of TmSm34/84, we also evaluated the inhibitory effect of TmSm34/84 on A549 CSCs. As shown in **Figure S4**, the viability of CSCs was markedly reduced in a dose-dependent manner when treated with TmSm34/84, and CSCs viability decreased by 7.32% when the concentration of TmSm34/84 was 1.05 μM. Given that non-cytotoxic dose is an important indicator for studying drug resistance reversal, TmSm34/84 of 1.05 μM was selected as a non-cytotoxic dose for further drug resistance reverse study (29). Compared with ADM treated alone group, TmSm34/84 (non-cytotoxic dose)-ADM combination group could significantly decrease the viability of CSCs (**Figure 10A**). The IC_50_ of CSCs to ADM was reduced from 10.58 to 1.51 μM, indicating that the reversal index of TmSm34/84 (non-cytotoxic dose) was 7.01. Annexin V-FITC/7-AAD experiment was further conducted to determine the effect of the combination of TmSm34/84 with ADM on CSCs apoptosis. As showed in **Figures 10B** and **S5**, the apoptotic rate of CSCs induced by the combination of TmSm34/84 with ADM was 1.93-fold than that of ADM treated alone. To detect the mechanism of TmSm34/84 (non-cytotoxic dose) reversed the drug resistance of CSCs, the expression levels of apoptosis-related protein (Survivin, Bcl-2, and cleaved caspase-3) and drug resistance-related protein (P-gp) in CSCs were analyzed by Western blot. Compared with ADM treated alone group, the expression levels of survivin, Bcl-2, and P-gp in the combination group were significantly decreased (P < 0.05), while cleaved caspase-3 expression was obviously increased (P < 0.05) (**Figure 10C**). The decreased expression of survivin, Bcl-2, and P-gp and the increased expression of cleaved caspase-3 may be contributed to the reversal of drug resistance of CSCs to ADM. The above results indicated that TmSm34/84 could not only inhibit the viability of CSCs, but also improve the sensitivity of CSCs to ADM remarkably.

## Discussions

Protein engineering is a technical means to introduce mutations in the amino acid sequence of target protein through genetic engineering, thus changing the spatial structure of the target protein and improving its function (30). There have been many examples for improving protein activity through protein engineering. For example, compared with monooxygenase P450pyr, P450pyr mutants were able to catalyze regioselective terminal hydroxylation of n-butanol to produce 1,4-butanediol, thus switching substrate acceptance of a hydroxylase from hydrophobic to hydrophilic compounds (31). Many enzyme mutants were obtained through iterative saturation mutagenesis (ISM), and most of these mutants were the best mutation collection which accumulated a series of beneficial mutation sites (32). But in this study, TmSm34/48/84, a three-site mutant protein which formed by combining three beneficial mutation sites (T34, T48, and C84), showed weaker pro-apoptotic capacity on A549 cells than that of TmSm34/84. This phenomenon was very interesting and only a few cases have been reported so far. For example, survivin mutant T48A could increase the affinity of survivin for borealin, thus inhibiting cell proliferation, while the introduction of a second mutation at T97 could alleviate this repression (17). This phenomenon embodies the truth that “a little wind kindles, much puts out the fire.” We believe that this phenomenon may be widespread in protein engineering. By analyzing the docking results of TmSm34/84 and TmSm34/48/84 with caspase-9 respectively, we found that TmSm34/48/84 had a higher interface area and a lower binding free energy value with caspase-9, which made it acquire a weaker capacity in inducing cell apoptosis than TmSm34/84. Hence in the rational design of protein engineering, it is not only necessary to create the number of beneficial mutations, but also should focus on the diversities of mutations. Rational analysis should carry out based on enzyme-substrate binding conformation so as to improve the characteristics of the mutants from different angles, thus obtaining performance match enzymes, rather than a multiple mutation sites superposition of a similar principle.

CSCs are cancer cells which have the characteristics of stem cells and the ability to self-replicate and multi-directional differentiation (33). CSCs account for only 1–2% of tumor cells, but they are considered to be the initiator of tumor occurrence and recurrence (27, 34). Traditional cancer therapies can kill most tumor cells, but have no effect on CSCs, which ultimately lead to tumor recurrence and stronger drug resistance acquiring (35, 36). Therefore, an appropriate chemoradiotherapy sensitizer should be found to enhance the sensitivity of CSCs to chemoradiotherapy so as to kill CSCs effectively. The combination of siRNA targeting survivin with ADM has been shown to reverse the resistance of breast CSCs to ADM. But the random integration and off-target of gene limited the application of gene therapy, so protein sensitizers might had a more significant advantage (37). Our previous studies have shown that TmSm34 could be used as a chemotherapy sensitizer to increase the sensitivity of breast cancer cells to ADM. However, whether TmSm proteins can be used as a chemotherapy sensitizer to improve the sensitivity of A549 CSCs to ADM has not been reported. In this study, we found that TmSm34/84 could inhibit the proliferation of CSCs and reverse the resistance of CSCs to ADM, indicating that the application of TmSm proteins with chemotherapy was a potentially effective treatment for cancer.

TmSm34 has been proved to be the most effective survivin mutant which could induce different degrees of apoptosis in various cancer cells and effectively inhibit tumor growth *in vivo* (15, 38). The cell specificities of survivin mutants were due to the natural difference in the expression abundance of survivin in different types of cancer cells. In this study, A549 cells with strong malignancy was used as the model cell line, and focused on exploring whether a survivin mutant protein with stronger anti-cancer activity could be obtained by adding one or two other mutation sites based on TmSm34. In summary, we successfully found a TmSm protein, TmSm34/84, which exhibited a potent ability in proliferation inhibition and apoptosis induction on A549 cells. Besides, it was also a potential protein drug as a chemosensitizer for A549 CSCs. And our further study will focus on testing the effects of TmSm proteins *in vivo*. In summary, these findings further promote the application of survivin dominant negative mutants in the clinical treatment of cancer and provide a new idea and method for solving the problem of drug resistance in the process of cancer treatment.

## Conclusions

This work showed that TmSm34/84 inhibited proliferation and induced apoptosis of A549 cells more efficiently than other TmSm proteins we prepared. Besides, TmSm34/84 could significantly elevate the sensitivity of A549 CSCs to ADM, with a reverse factor of 7.01. We further identified that the sensitization effect of TmSm34/84 on CSCs was produced by decreasing the expression levels of survivin, Bcl-2, and P-gp, thus eventually increasing the cleaved caspase-3 expression level. Overall, TmSm34/84 could potentially be a promising candidate drug for survivin-targeting cancer therapies.

## Data Availability Statement

The datasets presented in this study can be found in online repositories. The names of the repository/repositories and accession number(s) can be found in the article/**Supplementary Material**.

## Author Contributions

WG and WZ contributed equally to this work. WG designed and conducted the experiments totally and XM wrote the manuscript. WZ helped perform the analysis with constructive discussions. YF, CL, QL, FH, YL, and TZ also performed the experiments partly. FH, HM, and MH performed the data analyses and drawn the table. FY and YY revised the manuscript and gave some insightful suggestions. All authors contributed to the article and approved the submitted version.

## Funding

This study was supported by the National Key Research and Development Project of China (2018YFA0902804), the National Natural Science Foundation (31670944, 81673345), and the Science and Technology Innovation Action Plan of Shanghai (17431904600).

## Conflict of Interest

YY was employed by company SinGENE Biotech Pte Ltd.

The remaining authors declare that the research was conducted in the absence of any commercial or financial relationships that could be construed as a potential conflict of interest.

## References

[1] Bray F Ferlay J Soerjomataram I Siegel RL Torre LA Jemal A. Global cancer statistics 2018: GLOBOCAN estimates of incidence and mortality worldwide for 36 cancers in 185 countries. CA-CANCER J Clin (2018) 68(6):394–424. 10.3322/caac.21492 30207593

[2] Chi Y Wang D Wang J Yu W. Long non-coding RNA in the pathogenesis of cancers. Cells (2019) 8(9):1015. 10.3390/cells8091015 PMC6770362 31480503

[3] Harbeck N Penault-Liorca F Cortes J Gnant M Houssami N Poortmans P. Breast cancer. Nat Rev Dis Primers (2019) 5(1):66. 10.1038/s41572-019-0111-2 31548545

[4] Peery RC Liu JY Zhang JT. Targeting survivin for therapeutic discovery: past, present, and future promises. Drug Discovery Today (2017) 22(10):1466–77. 10.1016/j.drudis.2017.05.009 28577912

[5] Ebrahimiyan H Aslani S Rezaei N Jamshidi A Mahmoudi M. Survivin and autoimmunity; the ins and outs. Immunol Lett (2018) 193:14–24. 10.1016/j.imlet.2017.11.004 29155234

[6] Song Z Liu S He H Hoti N Wang Y Feng S. A single amino acid change (Asp53→ Ala53) converts survivin from anti-apoptotic to pro-apoptotic. Mol Biol Cell (2004) 15(3):1287–96. 10.1091/mbc.e03-07-0512 PMC363130 14699067

[7] Ejarque M Ceperuelo-Mallafré V Serena C. Survivin, a key player in cancer progression, increases in obesity and protects adipose tissue stem cells from apoptosis. Cell Death Dis (2017) 8(5):2802. 10.1038/cddis.2017.209 PMC5520726 28518147

[8] Wang T Gantier MP Xiang D Bean AG Bruce M Zhou SF. EpCAM aptamer-mediated survivin silencing sensitized cancer stem cells to doxorubicin in a breast cancer model. Theranostics (2015) 5(12):1456–72. 10.7150/thno.11692 PMC4672025 26681989

[9] Li F Aljahdali I Ling X. Cancer therapeutics using survivin BIRC5 as a target: what can we do after over two decades of study? J Exp Clin Cancer Res (2019) 38(1):368. 10.1186/s13046-019-1362-1 31439015 PMC6704566

[10] Zhang R Wang T Li KN Qin WW Chen R Wang K. A survivin double point mutant has potent inhibitory effect on the growth of hepatocellular cancer cells. Cancer Biol Ther (2008) 7(4):547–54. 10.4161/cbt.7.4.5484 18296918

[11] Coumar MS Tsai FY Kanwar JR Sarvagalla S Cheung CH. Treat cancers by targeting survivin: just a dream or future reality? Cancer Treat Rev (2013) 39(7):802–11. 10.1016/j.ctrv.2013.02.002 23453862

[12] Martinez-Garcia D Manero-Ruperez N Quesada R Korrodi-Gregorio L Soto-Cerrato V. Therapeutic strategies involving survivin inhibition in cancer. Med Res Rev (2019) 39(3):887–909. 10.1002/med.21547 30421440

[13] Ma X Zheng W Wei D Ma Y Wang T Wang J. High-level expression, purification and pro-apoptosis activity of HIV-TAT-survivin (T34A) mutant to cancer cells in vitro. J Biotechnol (2006) 123(3):367–78. 10.1016/j.jbiotec.2005.11.018 16406157

[14] Xu Y Zheng W Wang T Wang P Zhu L Ma X. Genetic protein TmSm(T34A) enhances sensitivity of chemotherapy to breast cancer cell lines as a synergistic drug to doxorubicin. BioMed Pharmacother (2012) 66(5):368–72. 10.1016/j.biopha.2011.12.004 22560635

[15] Ma X Zhang Y Kang Y Li L Zheng W. A recombinant protein TmSm(T34A) can inhibit proliferation and proapoptosis to breast cancer stem cells (BCSCs) by down-regulating the expression of Cyclin D1. BioMed Pharmacother (2016) 84:373–81. 10.1016/j.biopha.2016.08.066 27668537

[16] Cheung CH Sun X Kanwar JR Bai JZ Cheng L Krissansen GW. A cell-permeable dominant-negative survivin protein induces apoptosis and sensitizes prostate cancer cells to TNF-α therapy. Cancer Cell Int (2010) 10:36. 10.1186/1475-2867-10-36 20920299 PMC2958862

[17] Barrett RM Colnaghi R Wheatley SP. Threonine 48 in the BIR domain of survivin is critical to its mitotic and anti-apoptotic activities and can be phosphorylated by CK2 in vitro. Cell Cycle (2011) 10(3):538–48. 10.4161/cc.10.3.14758 PMC3115020 21252625

[18] Khan Z Khan AA Yadav H Prasad GBKS Bisen PS. Survivin, a molecular target for therapeutic interventions in squamous cell carcinoma. Cell Mol Biol Lett (2017) 22(1):1–32. 10.1186/s11658-017-0038-0 28536639 PMC5415770

[19] Shalini S Dorstyn L Dawar S Kumar S. Old, new and emerging functions of caspases. Cell Death Differ (2015) 22(4):526–39. 10.1038/cdd.2014.216 PMC4356345 25526085

[20] Lossi L Castagna C Merighi A. Caspase-3 mediated cell death in the normal development of the mammalian cerebellum. Int J Mol Sci (2018) 19(12):3999. 10.3390/ijms19123999 PMC6321612 30545052

[21] Chang JY Cheung CHA Huang CC Tsai FY Lee JY Cheng SM. Survivin - biology and potential as a therapeutic target in oncology. Onco Targets Ther (2013) 6:1453–62. 10.2147/ott.s33374 PMC3804542 24204160

[22] Sriramoju B Kanwar RK Kanwar JR. Nanoformulated cell-penetrating survivin mutant and its dual actions. Int J Nanomed (2014) 9:3279–98. 10.2147/IJN.S60169 PMC4099198 25045261

[23] Xue Z Sun PH Zhu LM Jiang SH Qiao MM Chi AL. Adeno-associated virus-mediated survivin mutant Thr34Ala cooperates with oxaliplatin to inhibit tumor growth and angiogenesis in colon cancer. Oncol Rep (2011) 25(4):1039–46. 10.3892/or.2011.1166 21279308

[24] Lee CH Yu CC Wang BY Chang WW. Tumorsphere as an effective in vitro platform for screening anti- cancer stem cell drugs. Oncotarget (2015) 7(2):1215–26. 10.18632/oncotarget.6261 PMC4811455 26527320

[25] Roudi R Madjd Z Ebrahimi M Samani FS Samadikuchaksaraei A. CD44 and CD24 cannot act as cancer stem cell markers in human lung adenocarcinoma cell line A549. Cell Mol Biol Lett (2014) 19(1):23–36. 10.2478/s11658-013-0112-1 24363164 PMC6275711

[26] Ning X Du Y Ben Q Huang L He X Gong Y. Bulk pancreatic cancer cells can convert into cancer stem cells (CSCs) in vitro and 2 compounds can target these CSCs. Cell Cycle (2016) 15(3):403–12. 10.1080/15384101.2015.1127471 PMC4943690 26709750

[27] Phi LTH Sari IN Yang YG Lee SH Jun N Kim KS. Cancer stem cells (CSCs) in drug resistance and their therapeutic implications in cancer treatment. Stem Cells Int (2018) 2018(28):1–16. 10.1155/2018/5416923 PMC5850899 29681949

[28] EC H. Postoperative chemotherapy for non-small-cell lung cancer. Chest (1993) 103(Suppl):30–4. 10.1378/chest.103.1.30S 8380131

[29] Nakasone ES Askautrud HA Kees T Park JH Plaks V Ewald AJ. Imaging tumor-stroma interactions during chemotherapy reveals contributions of the microenvironment to resistance. Cancer Cell (2012) 21(4):488–503. 10.1016/j.ccr.2012.02.017 22516258 PMC3332002

[30] MS F Whitehead T. Data-driven engineering of protein therapeutics. Curr Opin Biotechnol (2019) 60:104–10. 10.1016/j.copbio.2019.01.015 30822697

[31] Yang Y Chi YT Toh HH Li Z. Evolving P450pyr monooxygenase for highly regioselective terminal hydroxylation of n-butanol to 1,4-butanediol. Chem Commun (Camb) (2015) 51(5):914–7. 10.1039/c4cc08479a 25435422

[32] Yang Y Liu J Li Z. Engineering of p450pyr hydroxylase for the highly regio- and enantioselective subterminal hydroxylation of alkanes. Angew Chem Int Ed Engl (2014) 53(12):3120–4. 10.1002/anie.201311091 24554642

[33] Siddharth S Das S Nayak A Kundu CN. SURVIVIN as a marker for quiescent-breast cancer stem cells - an intermediate, adherent, pre-requisite phase of breast cancer metastasis. Clin Exp Metastas (2016) 33(7):661–75. 10.1007/s10585-016-9809-7 27411340

[34] Akbarzadeh M Maroufi NF Tazehkand AP Akbarzadeh M Bastani S Safdari R. Current approaches in identification and isolation of cancer stem cells. J Cell Physiol (2019) 6(1):178. 10.1002/jcp.28271 30741412

[35] Zhu F Qian W Zhang H Liang Y Wu M Zhang Y. SOX2 is a marker for stem-like tumor cells in bladder cancer. Stem Cell Rep (2017) 9(2):429–37. 10.1016/j.stemcr.2017.07.004 PMC5550032 28793245

[36] MacDonagh L Gray SG Breen E Cuffe S Finn SP O’Byrne KJ. Lung cancer stem cells: The root of resistance. Cancer Lett (2016) 372(2):147–56. 10.1016/j.canlet.2016.01.012 26797015

[37] Lee EJ Guenther CM Suh J. Adeno-associated virus (AAV) vectors: Rational design strategies for capsid engineering. Curr Opin BioMed Eng (2018) 7:58–63. 10.1016/j.cobme.2018.09.004 31106283 PMC6516759

[38] Ma X Zheng W Wei D Ma Y Wang T Wang J. Construction, expression, and purification of HIV-TAT-survivin (T34A) mutant: a pro-apoptosis protein in Escherichia coli. Protein Expr Purif (2006) 47(1):36–44. 10.1016/j.pep.2005.09.012 16260148

