# Supplementary material for: Discovering and Characterizing of Survivin Dominant Negative Mutants With Stronger Pro-apoptotic Activity on Cancer Cells and CSCs
Source: Front Oncol. 2021 Mar 31;11:635233. doi: 10.3389/fonc.2021.635233 (PMC8045750; doi:10.3389/fonc.2021.635233)
Supplement: Supplementary file 1 [file DataSheet_1.doc]

**Discovering and characterizing of****survivin dominant negative mutants with stronger pro-apoptotic activity on cancer cells and CSCs**

Wei Guo^1#^, Xingyuan Ma^1#^, Yunhui Fu^1^, Chang Liu^1^, Qiuli Liu^2^, Fabiao Hu^1^, Hui Miao^1^, Tong Zhang^2^, Yuping Liu^2^, Myong Hun Han^1,3^, Fang You^4^, Yi Yang^4,5^*, Wenyun Zheng*^2^

*^1^State Key Laboratory of Bioreactor Engineering, East China University of Science and Technology, Shanghai 200237, P. R. China*

*^2^Shanghai Key Laboratory of New Drug Design, School of Pharmacy, East China University of Science and Technology, Shanghai 200237, P. R. China*

*^3^Department of Genetics, Faculty of Life Science, KIM IL SUNG University, Pyongyang 999093, Democratic People’s Republic of Korea*

*^4^Department of Chemical and Biomolecular Engineering, National University of Singapore, Singapore 117585, Singapore*

*^5^**SinGENE Biotech Pte Ltd, Singapore Science Park, Singapore 118258, Singapore*

**Supplementary Materials**

**Supplementary Figures:**


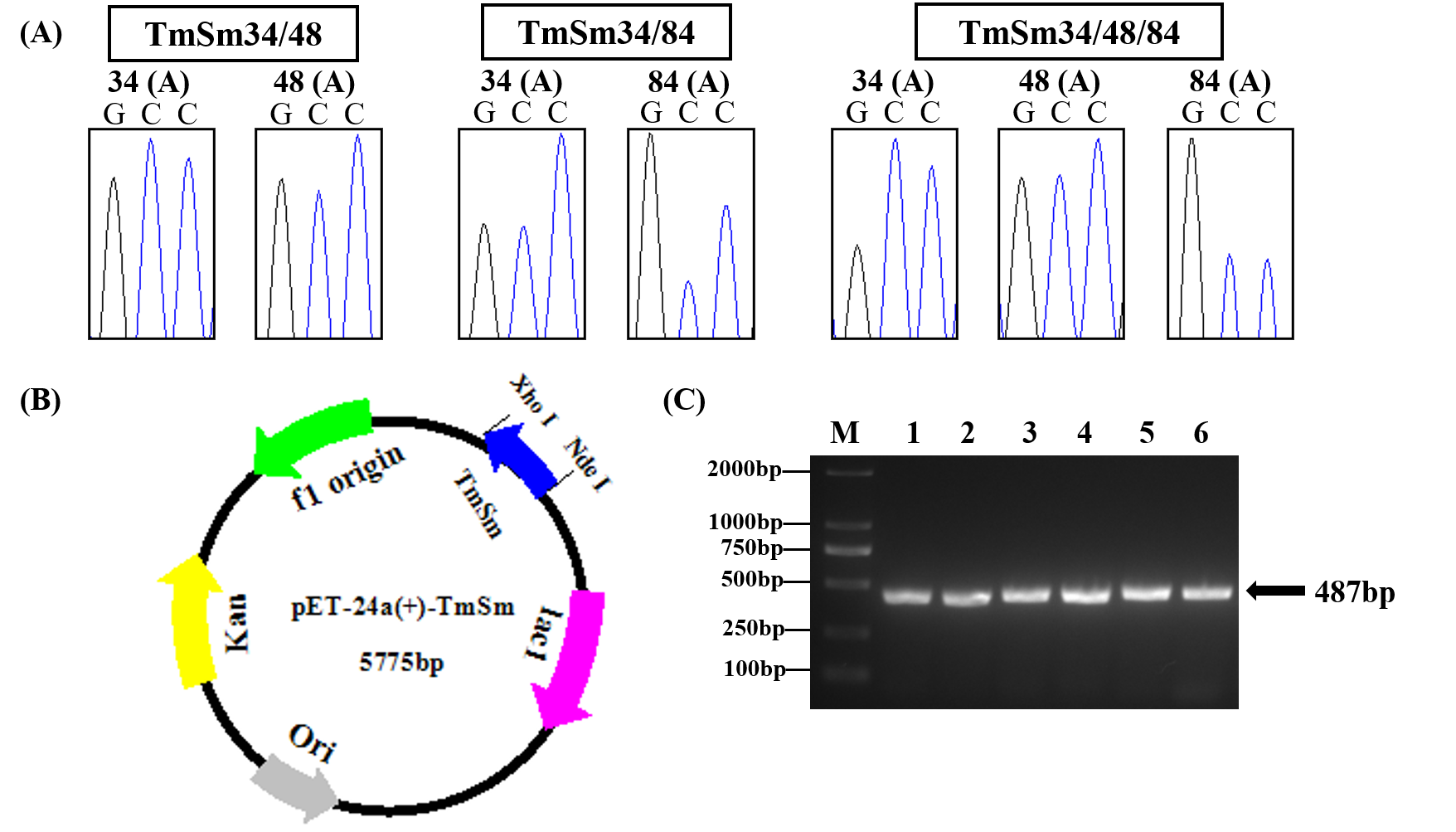


**Figure S1. Construction of recombinant plasmids.** (A) DNA sequencing of survivin mutants. (B) Schematic diagram of the construction of recombinant plasmids. (C) Agarose gel electrophoresis of survivin mutants via PCR amplification. Lane M, DL2000 marker; Lane 1-2, TmSm34/48 amplification fragment; Lane 3-4, TmSm34/84 amplification fragment; Lane 5-6, TmSm34/48/84 amplification fragment.


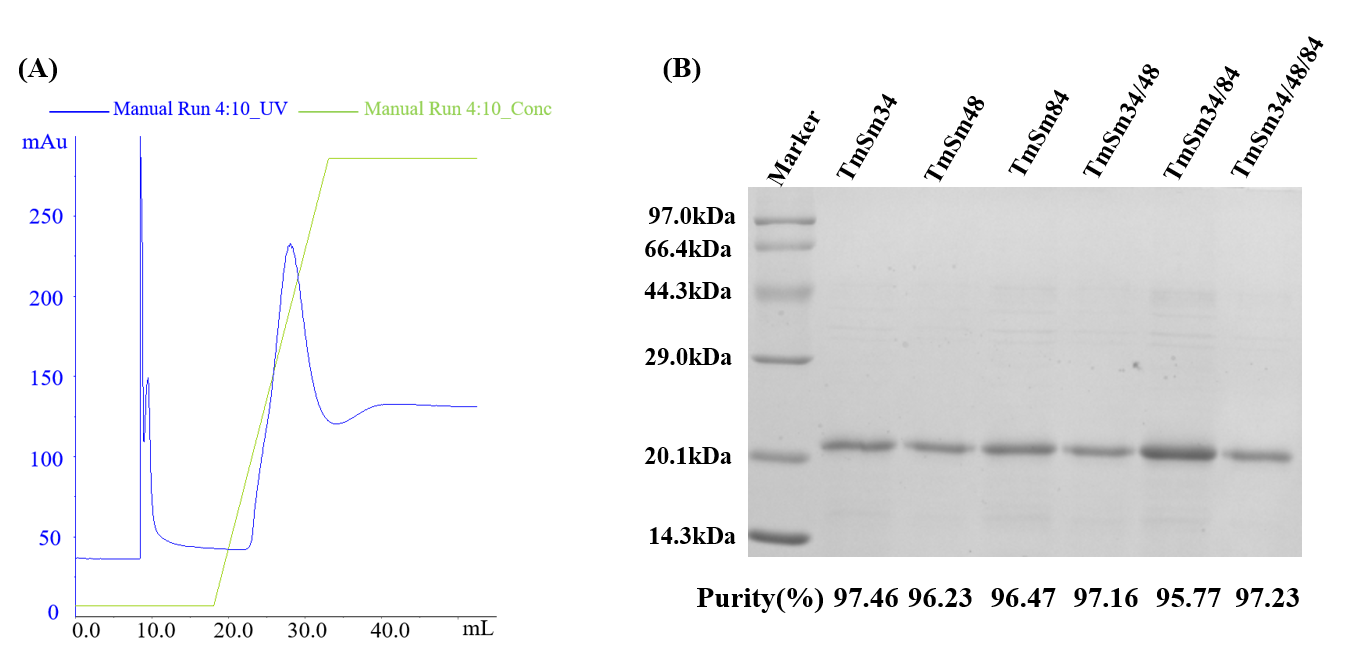


**Figure S2. Purification of TmSm proteins and SDS-PAGE analysis.** (A) Purification of TmSm proteins *via* nickel column chromatogram. (B) The purified TmSm proteins were analyzed by SDS-PAGE.


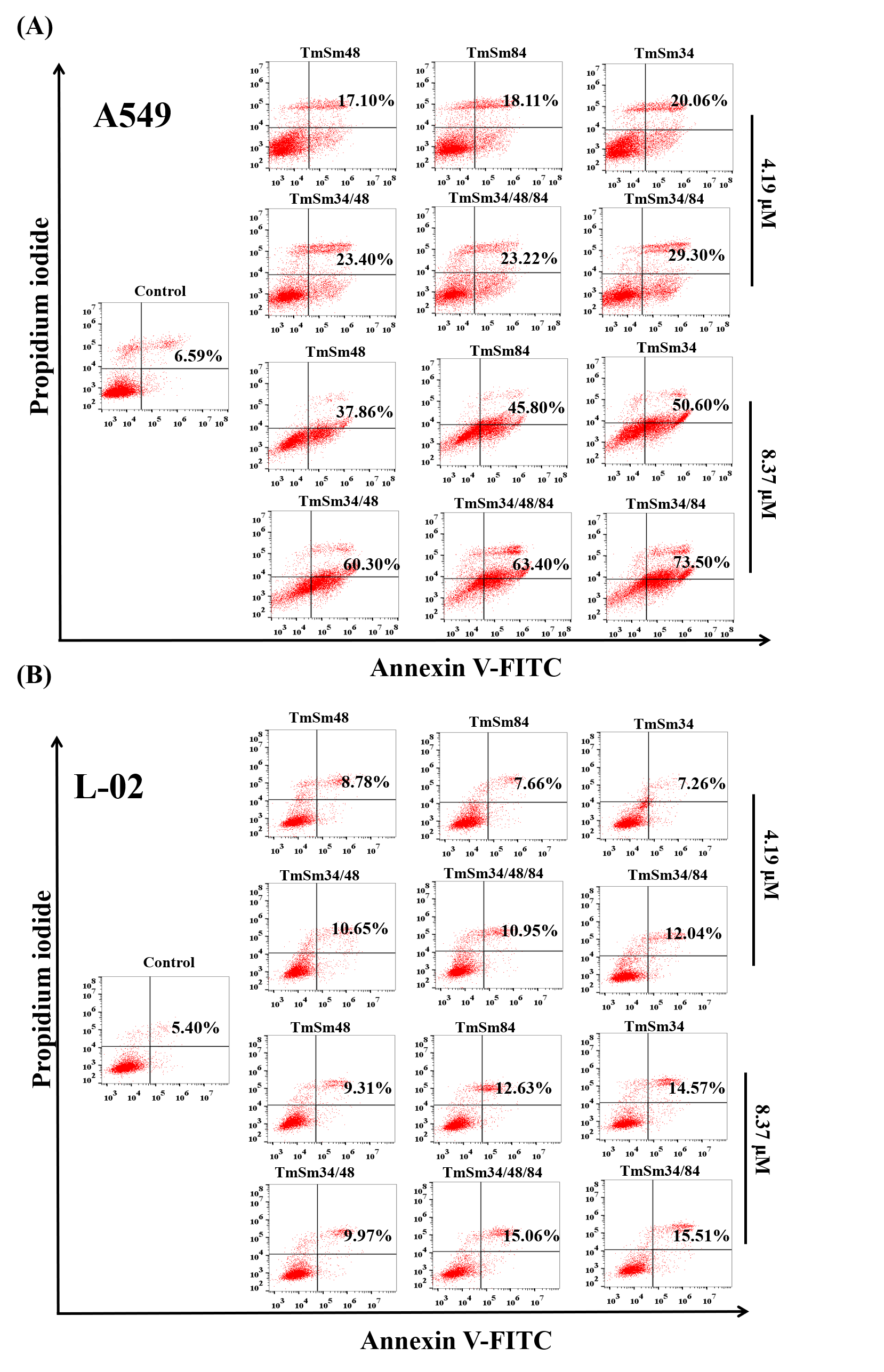


**Figure S3. Apoptosis of A549 and L-02 cells were analyzed by flow cytometry.** (A) A549 cells were incubated with different TmSm proteins of the same concentrations gradient (0, 4.19, and 8.37 μM) for 24 h. The apoptosis rate was analyzed by flow cytometry. (B) L-02 cells were incubated with different TmSm proteins of the same concentrations gradient (0, 4.19, and 8.37 μM) for 24 h. The apoptosis rate was analyzed by flow cytometry. Data were expressed as mean ± SD (n = 3). *P < 0.05, **P < 0.01, and ***P < 0.001.


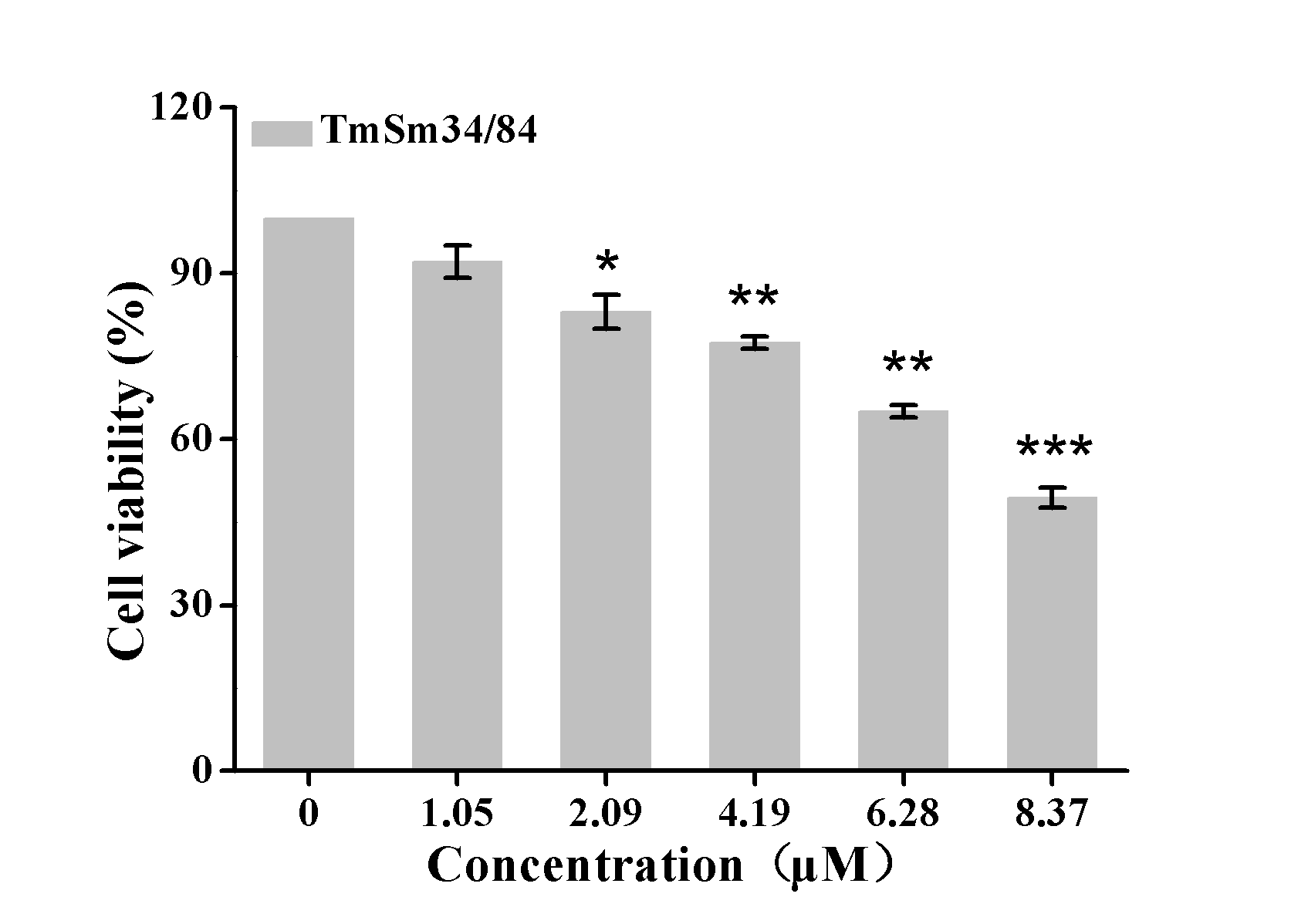


**Figure S4. Cytotoxicity assay of TmSm34/84 against CSCs.** The viability of CSCs was measured by incubating with TmSm34/84 of different concentrations (0, 2.09, 4.19, 6.28, and 8.37 μM) for 24 h. Data were expressed as mean ± SD (*n* = 3). ***P* < 0.01 and ****P* < 0.001.


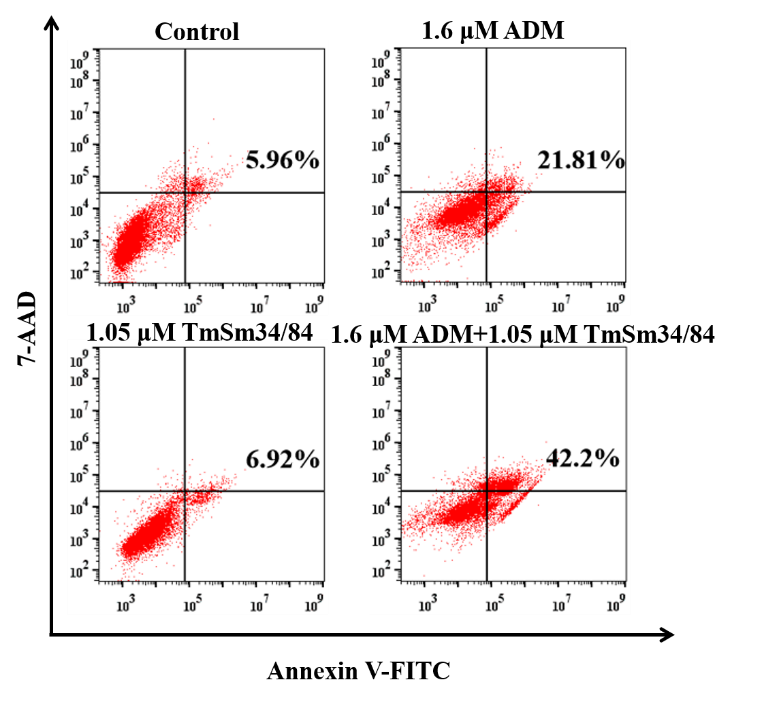


**Figure S5. Apoptosis of CSCs was analyzed by flow cytometry.** CSCs were incubated with ADM (1.6 μM), TmSm34/84 (1.05 μM) and their combination, respectively. The apoptosis rate was analyzed by flow cytometry.

**Tables:**

**Table S1. Information of different primers used for the production of TmSm genes.**

| Gene | Fragment | Primer | Sequence (5’-3’) | Size (bp) |
| --- | --- | --- | --- | --- |
| TmSm  34/48 | First | Forward | GGAATTCCATATGTACGCTCGTAAAGCTCGT | 210 |
|  |  | Reverse | TCTGGCTCGTTCTCGGCGGGGCAGTGGATG |  |
|  | Second | Forward | CATCCACTGCCCCGCCGAGAACGAGCCAGA | 307 |
|  |  | Reverse | CCGCTCGAGATCCATGGCAGCCAGCTGCTCG |  |
| TmSm  34/84 | First | Forward | GGAATTCCATATGTACGCTCGTAAAGCTCGT | 317 |
|  |  | Reverse | CAGAAAGGAAAGCGGCACCGGACGAATGCT |  |
|  | Second | Forward | AGCATTCGTCCGGTGCCGCTTTCCTTTCTG | 200 |
|  |  | Reverse | CCGCTCGAGATCCATGGCAGCCAGCTGCTCG |  |
| TmSm  34/48/84 | First | Forward | GGAATTCCATATGTACGCTCGTAAAGCTCGT | 316 |
|  |  | Reverse | AGAAAGGAAAGCGGCACCGGACGAATGCTT |  |
|  | Second | Forward | AAGCATTCGTCCGGTGCCGCTTTCCTTTCT | 201 |
|  |  | Reverse | CCGCTCGAGATCCATGGCAGCCAGCTGCTCG |  |

**Table S2. The yield and purity of TmSm proteins in different treatment stages.**

| Stage | Sonication | | Wash | | Purification | | Renaturation | |
| --- | --- | --- | --- | --- | --- | --- | --- | --- |
| Protein | Yield (%) | Purity  (%) | Yield (%) | Purity  (%) | Yield (%) | Purity  (%) | Yield (%) | Purity  (%) |
| TmSm34 | 100 | 68.15±5.23 | 76.50±1.26 | 79.05±3.12^a^ | 56.12±2.03 | 97.46±2.32^a^ | 30.12±2.43 | 96.79±2.15^a^ |
| TmSm48 | 100 | 69.42±1.19 | 74.31±3.69 | 78.28±4.05^a^ | 56.56±1.89 | 96.23±2.57^a^ | 35.24±4.26 | 98.44±2.19^a^ |
| TmSm84 | 100 | 71.19±3.61 | 75.24±2.27 | 77.28±0.81^a^ | 57.34±2.76 | 96.47±3.58^a^ | 33.17±1.67 | 96.65±3.26^a^ |
| TmSm34/48 | 100 | 71.57±1.14 | 78.32±2.10 | 78.88±2.21^a^ | 58.42±1.23 | 97.23±2.14^a^ | 34.67±3.20 | 97.15±3.59^a^ |
| TmSm 34/84 | 100 | 69.44±3.40 | 76.35±3.47 | 75.15±4.74^a^ | 56.31±3.54 | 95.77±2.54^a^ | 36.47±1.76 | 95.24±3.69^a^ |
| TmSm 34/48/84 | 100 | 67.34±2.12 | 75.23±3.21 | 76.42±  1.35^a^ | 57.69±  2.14 | 97.23±  1.67^a^ | 37.45±1.28 | 96.47±  2.26^a^ |

The data were expressed as the mean ± SD (*n* = 3). ^a^*P* < 0.05 compared with sonication group.

**Table S3. The IC_50_ values of different TmSm proteins against L-02 and A549 cells.**

| Cells | IC_50_(μM) | | | | | |
| --- | --- | --- | --- | --- | --- | --- |
|  | TmSm  48 | TmSm  84 | TmSm  34 | TmSm  34/48 | TmSm  34/48/84 | TmSm  34/84 |
| L-02 | 52.22 ± 3.24 | 51.68 ±  2.12 | 25.04±  1.36 | 42.03 ±  4.78 | 42.23 ± 2.24 | 36.82±  3.65 |
| A549 | 11.83 ± 2.13^a^ | 10.2 ±  1.54^a^ | 9.44±  1.35^a^ | 6.86 ±  0.89^b^ | 6.32 ± 0.76^b^ | 5.51±  0.94 |

The data were expressed as the mean ± SD (*n* = 3). ^b^*P* < 0.05 and ^a^*P* < 0.01 compared with TmSm34/84.
